# Primary Care Spending in the Veterans Health Administration in 2014 and 2018

**DOI:** 10.1001/jamanetworkopen.2021.17533

**Published:** 2021-07-19

**Authors:** Ashok Reddy, Karin M. Nelson, Edwin S. Wong

**Affiliations:** 1 Center of Innovation for Veteran-Centered and Value-Driven Care, VA Puget Sound Health Care System, Seattle, Washington; 2 Department of Health Services, University of Washington, Seattle; 3 Division of General Internal Medicine, Department of Medicine, University of Washington, Seattle

## Abstract

This quality improvement study assesses the proportion of primary care spending in the Veterans Health Administration in 2014 and 2018.

## Introduction

Although primary care is associated with higher quality of care, better outcomes, and lower costs, a low proportion is spent on primary care in the US among both commercial payers (4%-8%) and Medicare fee-for-service (2%-5%).^1,2^ The Veterans Health Administration (VHA), one of the largest integrated health systems, has made major investments in primary care over the past decade.^3^ However, to our knowledge, resources spent on primary care in the VHA have not been previously estimated. In this quality improvement study, we assess the proportion of primary care spending in the VHA in 2014 and 2018.

## Methods

 This evaluation was reviewed and designated as nonresearch quality improvement by the VHA Office of Primary Care and, therefore, did not require institutional review board approval or informed consent, in accordance with 45 CFR §46. This study followed the Strengthening the Reporting of Observational Studies in Epidemiology (STROBE) reporting guideline.

We used 2 data sources for analysis: VHA administrative files and the Health Economics Resource Center Average Cost files. Encounter-level costs are based on Health Economics Resource Center estimates that are proportional to Medicare reimbursement rates for similar encounters. Pharmacy costs include drug fees and dispensing costs. We calculated total VHA spending in fiscal years 2014 and 2018 by 6 categories: primary care and integrated behavioral health, specialty mental health, medical and surgical outpatient care (eg, cardiology, pulmonology, or urology), inpatient care (eg, medical, surgical, postacute care, or emergency department), pharmacy, and diagnostic or other spending (eg, laboratory or radiology). Outpatient care categories were derived from VHA-specific stop code groups.^3^ Primary care services were defined using stop code groupings identified in prior research,^3^ reflecting clinic locations considered to have provided primary care. For each year, we summed costs for all encounters within the 6 categories. We converted 2014 estimates to 2018 constant dollars using the Consumer Price Index. Finally, we calculated the percentage of total costs considered primary care by age, sex, and race. Race was assessed in this study to inform whether primary care resources are reaching veterans from marginalized and underserved groups. Race was ascertained from patient-level records in the Observational Medical Outcomes Partnership Common Data Model. Data were analyzed using SAS Enterprise Guide statistical software version 8.3 (SAS Institute) from July 2020 to March 2021.

## Results

Among more than 6 million veterans in 2014 and 2018, the median (interquartile range) age was 65 (50-73) years, and a majority were male (5 438 239 men in 2014 and 5 499 978 men in 2018 [90%]) and White (4 260 547 individuals in 2014 and 4 462 266 individuals in 2018 [71%]) (Table 1). In 2014, total spending in the 6 categories was $50.9 billion. Primary care represented 9% of this total. Inpatient (36%), medical and surgical outpatient care (21%), pharmacy (13%), and diagnostic or other (13%) each represented a larger share of total spending compared with primary care (Table 2). By 2018, total spending increased to $58.5 billion, and primary care accounted for 8% of this total.

**Table 1.** Total and Primary Care Spending by Selected Demographic Characteristics, 2014 and 2018

Characteristic	2014		2018	
	Total No. of enrollees^a^	Total spending, $ (% primary care)	Total No. of enrollees^a^	Total spending, $ (% primary care)
Age, y				
<40	796 590	4 539 520 892 (10.5)	929 450	6 075 898 578 (9.3)
40-54	1 008 381	9 986 746 974 (7.5)	1 004 408	10 431 030 408 (6.8)
55-64	1 283 912	18 225 642 151 (6.0)	1 090 842	17 789 206 328 (4.8)
65-74	1 637 585	19 041 752 584 (7.0)	1 853 443	27 946 660 429 (5.8)
≥75	1 295 446	10 665 290 890 (7.6)	1 280 935	13 167 997 511 (6.1)
Sex				
Female	583 782	4 673 868 090 (8.5)	658 920	6 272 176 387 (7.5)
Male	5 438 239	57 790 206 572 (7.0)	5 499 978	69 191 638 427 (5.9)
Race				
American Indian or Alaska Native	40 909	469 666 618 (7.1)	47 094	574 874 922 (6.1)
Asian	54 401	351 446 349 (11.3)	67 141	518 436 094 (10.0)
Black or African American	946 985	12 947 901 701 (6.3)	1 059 062	16 513 675 762 (5.3)
Native Hawaiian or Pacific Islander	47 591	462 855 941 (8.3)	54 220	624 122 435 (6.7)
Unknown	671 649	3 884 728 681 (8.5)	650 573	4 251 743 061 (7.6)
White	4 260 547	44 347 842 705 (7.2)	4 462 266	53 192 923 352 (6.1)

^a^ Calculations exclude patients in missing categories.

**Table 2.** Total and Proportion of Spending in 6 Categories in Veterans Health Administration, 2014 and 2018

Category	Spending, $ (%)	
	2014	2018
Primary care	4 749 083 010 (9)	4 572 875 325 (8)
Inpatient care	18 498 203 553 (36)	19 650 618 393 (34)
Medical and surgical outpatient care	10 859 923 429 (21)	14 388 993 692 (24)
Pharmacy	6 593 706 620 (13)	8 117 466 013 (14)
Diagnostic or other	6 434 280 992 (13)	7 203 285 343 (12)
Specialty mental health	3 778 565 672 (8)	4 525 060 207 (8)

Primary care spending varied across patients’ demographic characteristics (Table 1). In 2014, primary care spending ranged from 6.0% among patients aged 55 to 64 years to 10.5% among patients younger than 40 years. The percentage of spending within primary care was higher for female vs male patients (8.5% vs 7.5%). Variation also emerged by race with the lowest percentage of primary care spending among Black patients (6.3%) and the highest percentage of spending among Asian patients (11.3%). Consistent with overall estimates, the proportion spent on primary care decreased for all demographic groups between 2014 and 2018.

## Discussion

The proportion of primary care spending in the VHA was low in both 2014 and 2018. In addition, we found substantial variation in primary care spending by veterans’ demographic characteristics. Although direct comparison to other systems differs because of methods, our estimates suggest that primary care spending by the VHA may be higher than that by other US payers but lower than that by health systems outside the US (12%-17% in Organisation for Economic Co-operation and Development countries).^4^ In addition, our estimates are below what many states have set as targets (>10% of spending on primary care) to improve outcomes and lower total costs of care.^5^ Study limitations include that veterans may receive care outside the VHA. Current estimates of costs of care obtained outside the VHA range from $8.2 billion in 2014 to $14.9 billion in 2018.^6^ Approximately 30% is spent on outpatient services, but specific data for primary care spending are unknown.^6^ Our results suggest opportunities to increase investment in primary care to improve care for veterans.

## References

[1] Reid R, Damberg C, Friedberg MW. Primary care spending in the fee-for-service Medicare population. JAMA Intern Med. 2019;179(7):977-980. doi:10.1001/jamainternmed.2018.8747 30985864 PMC6583869

[2] Reiff J, Brennan N, Fuglesten Biniek J. Primary care spending in the commercially insured population. JAMA. 2019;322(22):2244-2245. doi:10.1001/jama.2019.16058 31821423 PMC7081755

[3] Hebert PL, Liu CF, Wong ES, . Patient-centered medical home initiative produced modest economic results for Veterans Health Administration, 2010-12. Health Aff (Millwood). 2014;33(6):980-987. doi:10.1377/hlthaff.2013.0893 24889947

[4] Baillieu R, Kidd M, Phillips R, . The Primary Care Spend Model: a systems approach to measuring investment in primary care. BMJ Glob Health. 2019;4(4):e001601. doi:10.1136/bmjgh-2019-001601 31354975 PMC6626519

[5] Martin S, Phillips RL Jr, Petterson S, Levin Z, Bazemore AW. Primary care spending in the United States, 2002-2016. JAMA Intern Med. 2020;180(7):1019-1020. doi:10.1001/jamainternmed.2020.1360 32421142 PMC7235902

[6] US Government Accountability Office. VA health care: estimating resources needed to provide community care (GAO-19-478). Published June 12, 2019. Accessed June 15, 2021. https://www.gao.gov/products/gao-19-478

